# How does asthma influence the daily life of children? Results of focus group interviews

**DOI:** 10.1186/1477-7525-8-5

**Published:** 2010-01-14

**Authors:** Lisette van den Bemt, Sabine Kooijman, Vinca Linssen, Peter Lucassen, Jean Muris, Gordon Slabbers, Tjard Schermer

**Affiliations:** 1 Department of Primary and Community Care. Centre for Family Medicine, Geriatric Care and Public Health, Radboud University Nijmegen Medical Centre, the Netherlands; 2 FC Donders Centre for Cognitive Neuroimaging, Radboud University Nijmegen, the Netherlands; 3 Mondriaan zorggroep, Heerlen, the Netherlands; 4 Department of General Practice, Research institute Caphri, Maastricht University, the Netherlands; 5 Department of Pediatrics, Bernhoven hospital, Oss, the Netherlands

## Abstract

**Background:**

Health-related quality of life (HRQL) brings together various aspects of an individual's subjective experience that relate both directly and indirectly to health, disease, disability, and impairment. Although asthma is the most common chronic disease in childhood, information on pediatric patients' views on asthma-specific HRQL has not been described before. The aim of this study was to establish the components of asthma-specific HRQL, as experienced by primary school-aged asthmatic children. The generated components will be used to develop an individualized HRQL instrument for childhood asthma.

**Methods:**

Primary school-aged asthmatic children were invited to participate in three consecutive focus group sessions. A total of five focus groups were formed. Two reviewers independently 1) identified trends in the statements and relations between HRQL components, 2) clustered the components into a small number of domains and, 3) made a model on asthma-specific HRQL based on the transcribed statements of the children. The results were compared between the two reviewers and resulted in a final model.

**Results:**

Asthma influenced the life of the children physically, emotionally and socially. The most important components of HRQL were the effects on, and consequences of asthma on peer relationships (e.g., being bullied), the dependence on medication, shortness of breath, cough, limitations in activities and limitations due to the response on cigarette smoke exposure.

**Conclusion:**

The outcome of the focus group meetings indicates that asthma influences the life of children in various ways. Not all essential components of HRQL, according to the children, are part of existing asthma-specific HRQL instruments.

## Background

Dyspnea, dependence of medication, and not being able to fully integrate with peers are among the many aspects that could negatively influence the life of asthmatic children. Health-related quality of life (HRQL) brings together various aspects of an individual's subjective experience that relate both directly and indirectly to health, disease, disability, and impairment[1]. Since HRQL is a uniquely personal perception, the individual's view on the components of asthma-specific HRQL is the preferred basis of a content-valid HRQL instrument [2,3]. For asthma, several self-administered questionnaires to assess disease-specific HRQL in primary school-aged children with asthma have been developed, the most prominent ones being the *Pediatric Asthma Quality of Life Questionnaire *(PAQLQ) [4], the *How Are You *(HAY) instrument [5], The *Pediatric Quality of Life Inventory (PEDsQL™) Generic Core Scales and Asthma Module *[6], and the *Childhood Asthma Questionnaire *(CAQ-B) [7]. The agreement on HRQL components between these questionnaires is rather low: only some HRQL components of the symptoms domain and activity limitations domain are part of all questionnaires [8-11]. This is striking, when one realizes that all instruments were developed to measure the same concept. Do the questionnaires actually include all relevant aspects of disease-specific HRQL for children with asthma? The content validity of an instrument is influenced by the item selection procedure used to develop the questionnaire [12]. Focus group methodology is especially useful to determine children's ideas regarding HRQL, and though some published papers may suggest otherwise, this information is currently lacking[13]. In this paper we report findings from a series of focus group interviews with primary school-aged children with asthma, conducted to establish the components of asthma-specific HRQL according to the children themselves. The generated components will be used to develop an individualized HRQL instrument for childhood asthma. Individualized instruments are designed to detect individuals' problems and provide relevant information for clinical practice, while all available asthma specific HRQL instruments, so far, serve research purposes primarily.

## Methods

### Study participants

We invited children for participation via three general practices and one hospital pediatric outpatient clinic. The medical ethics review board Arnhem - Nijmegen approved the study. Informed consent was obtained from the parents before any study procedure took place. Children received information on the project, adjusted to their developmental stage prior to the first focus group meeting, that stressed the importance of voluntary participation. The moderators were instructed to withdraw children from the study when doubt was raised about the willingness of children to participate. Inclusion criteria for study participation were: (1) having asthma, defined as being diagnosed with asthmatic disease by a physician and having asthmatic complaints, like wheezing, dyspnea, and cough in the last year, requiring treatment with inhaled corticosteroids and/or bronchodilators (reported by the parents); and (2) aged between 6 and 12 years. Exclusion criteria were: (1) serious morbidity other than asthma that influenced HRQL; (2) too easily distracted to participate in focus group sessions; and (3) not being able to attend a regular school class. Information on asthmatic complaints, medication use, and exclusion criteria were collected by a questionnaire that was filled out by the parents after informed consent was obtained. Purposive sampling - maximum variation sampling - was used to assure maximum variation in disease severity and age within the study population. For that purpose, the children were selected using stratification by age (6 to 9 years *versus *9 to 12 years) and by asthma severity (intermittent and mild disease *versus *moderate to severe disease, according to the GINA guidelines) [14]. Homogeneity within a group allows children to share their experiences [15].

A priori we considered 4 focus groups of 5 children each to be sufficient to reach information saturation on components of HRQL in childhood asthma: i.e., to reach a state in which no additional insights on the subject matter were obtained by the investigators. We anticipated in planning additional focus groups when new items would still arise in the final focus group.

### Semi-structured focus group

The participating children joined the focus group session at three separate occasions within a 2-week period. The maximum duration of each session was 60 minutes, including a short halftime break. All focus group sessions took place at a primary school in the neighborhood of the child. The parents were not present during the group discussions. All sessions were digitally audiotaped for analysis. A list of topics to be discussed was constructed in an expert panel (including a pediatric psychologist, a parent of a child with asthma, a mental health scientist, a pediatric pulmonologist, an epidemiologist, a health scientist, and two family practitioners). The topic list was tested in a pilot focus group of asthmatic children. We used a funnel-based interview: in other words, each group starts with a less structured approach that emphasizes free discussion and then moves toward a more structured discussion of specific questions [16]. In this study, it meant that children were able to mention components of HRQL spontaneously and subsequently later on domains, and components of HRQL were probed to collect information on those issues that were not mentioned by the children previously. Throughout this paper, the term component is used to refer to an aspect that relates directly or indirectly to asthma-specific HRQL, while a domain refers to a cluster of components that cover a specific area of HRQL. The issues that were probed were considered potential components of HRQL, because they were 1) items of developed questionnaires, or 2) a component of HRQL according to literature or expert opinion. These components were divided in five domains, namely symptoms, limitations in activities, impact on social life, emotional impact, and cognitive impact. All issues that were mentioned by the children were considered as components of HRQL, irrespectively whether components were part of the list of potential components or not.

Two certified developmental psychologists guided the focus group sessions, alternately as the moderator or the observer. In the first session of each group, the moderator introduced the topic of the sessions with a chapter of a children's book of the Dutch Asthma Foundation about a girl with asthma [17]. Next, the children were asked to make a drawing about something they felt was related to their asthma. The moderator used the drawings for the facilitation of the discussion during the first session [18,19]. In the second session, the discussion was facilitated by a fishing rod game with visual cues of the five domains of HRQL to introduce different dimensions of HRQL [19]. Based on domain cards that were fished out by the children, children were asked to think about HRQL issues that were related to these domains. When children mentioned components that could be related to other diseases (e.g., sore throat, and limitations due to seasonal changes), the moderator asked if they were related to asthma. Next, the moderator probed items that were part of the component list and were not voluntarily mentioned by the children. After the children agreed that all relevant items had been discussed, the moderator started with the nominal group technique (NGT) in the third session [20]. Instead of the traditional voting procedure used in the NGT, the moderator asked the children to imagine that they had magical powers and could make the aspects disappear that they disliked most about their asthma. After each child had selected the three worst aspects of asthma, the children received a wizard hat in turn and told the selected issues.

During all focus group meetings, the moderator visualized the components of HRQL that were mentioned by the children on a large plastic-coated poster. If the children mentioned a new component, an accompanying pictogram was added to the poster.

### Data analysis and presentation

Immediately after each session, the audiotapes were transcribed and analyzed by the researchers, thus providing the opportunity to detect gaps, reveal unclear comments, and facilitate the rolling interview guide for the upcoming session of the same focus group. This approach provided the moderators with essential information from the previous sessions and recommendations for the upcoming session. Two reviewers (LB and SK) independently coded all statements of the children concerning components of HRQL. Some statements were coded twice, because they provided information on more than one component. When a child emphasized a particular component for the second time, this statement was coded again. Consequently, we were able to calculate the total number of statements per code. In case of discordance between the two reviewers, a third reviewer (TS) was consulted.

The outcome of the NGT was used to weigh the importance of the finally identified components. The components that had been labeled as the most important for the children received 3 points, the second-most important component received 2 points, and the third-most important component received 1 point. A total score per component was calculated.

**Figure 1:**
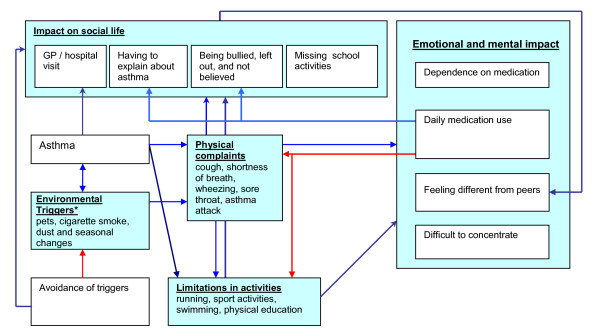
**Model of most prominent HRQL components and interactions**. The shaded text boxes are the domains on childhood asthma-specific HRQL. The arrows describe the relation between domains and components. If an arrow ends at the border of a domain, the aspect is related to the whole domain. If the arrow ends at a component, than the aspect is related to the specific component. Blue arrows represent a positive relation, red arrows represent an inhibiting relation.

For a more detailed textual analysis of the data, we used Atlas.ti software (Version Win 4.2, Scientific Software Development, Berlin Germany). The software enabled the reviewers to search all text transcripts for specific content information. Two reviewers independently attempted to unveil trends in the statements and relations between components, to cluster the components into a small number of domains, and to construct a model on asthma-specific HRQL based on statements of the children [16]. In this qualitative data analysis, the reviewers' judgment on components of HRQL was not only based on the number of statements, but the level of impairment mentioned by the children, the importance of the component emphasized by the children, and the number of children that had mentioned the component were also taken into account. The two reviewers summarized and compared trends and clusters and visualized the relations between the components of HRQL in a graphical model (Figure 1). In case of disagreement between the two reviewers, a third reviewer was consulted. The final results of the data analysis were presented to the developmental psychologists that guided the sessions to verify if the final model reflected a good view on the outcome of the focus group meetings (face-validity).

Narrative descriptions of the results per domain are presented in the Results section. In this narrative description, some illustrating quotes are provided. All information in the Results section was based on statements made by the children in the focus group sessions. In this article, all HRQL described is asthma-specific HRQL, except when stated otherwise.

## Results

### Study participants

**Figure 2:**
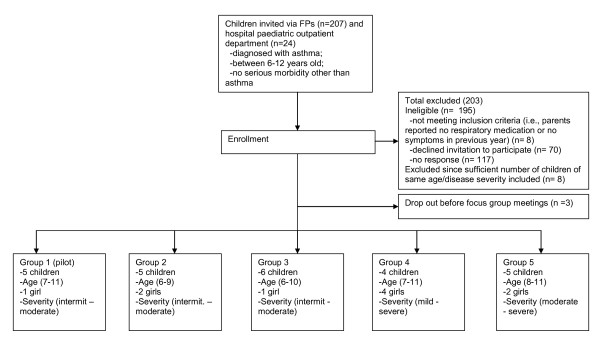
Recruitment of participants

A total of 231 families were invited to let their child with asthma participate in the focus group study. The selection of children is given in Figure 2. Parents' main reason to decline participation was that they did not consider their child to suffer from asthma anymore (76%). Moreover, 5 children were not willing to participate, and 8 children were excluded because sufficient numbers of children of the same age and disease severity had already been included. No children were excluded because the child was too easily distracted or was not able to attend a regular school class. All families were represented by only one child (i.e., no siblings participated). Table 1 shows that age categories and disease severity levels were well balanced in the study population. Some children did not attend all sessions for personal reasons (i.e., sickness/hospital visits). Finally, 5 focus groups with a size of 4-6 children were formed. The fifth focus group did not result in components that were not mentioned by the former focus groups, and therefore, information saturation was achieved.

**Table 1:** Characteristics of the children in the focus groups (n = 25)

Age, mean (range)	8.5 (6 - 11)
Gender	
Males (%)	16 (64)
GINA category asthmatic disease^1^, n (%)	
Intermittent	6 (24)
Mild	6 (24)
Moderate	9 (36)
Severe	4 (16)
Educational level mother^2^, n (%)	
Low	7 (28)
Middle	14 (56)
High	3 (12)
Missing	1 ( 4)
Educational level father^2^, n (%)	
Low	7 (28)
Middle	5 (20)
High	12 (48)
Unknown	1 ( 4)
Family structure, n (%)	
2 parents	21 (84)
Single parent	4 (16)

^1 ^GINA: Global Initiative for Asthma (10); ^2 ^education level was classified as low (pre-primary, primary, and lower secondary education), middle (upper secondary education), and high (pre-university education, higher vocational education, and university)

### Components of HRQL

The two reviewers identified a total of 868 unique HRQL statements in the focus group transcripts. Based on our data analyses, the components of HRQL were divided in the following 5 domains: limitations in activities (122 statements about limitations in activities were brought up in the focus group meetings), asthmatic symptoms (comprising 181 statements), impact on social life (comprising 259 statements), limitations due to environmental triggers (comprising 117 statements), and mental and emotional impact of asthmatic disease(comprising 189 statements). The mental and emotional domain also contained the use of medication. Table 2 is a list of all items mentioned by the children per focus group. The number of statements of the most important components of HRQL according to the reviewers are given in Table 3.

**Table 2:** List of items mentioned by children by focus group

Domain	Component	Focusgroup
*Limitations due to environmental triggers*		

	**No pets^1^**	1,2,3,4,5
	**Cigarette smoke**	1,2,3,4,5
	**Dust (house dust mite)**	1,2,3,4,5
	**Seasonal changes**	1,3,4,5
	Strong smelling substances like perfume	1,2,3
	Changes in the weather	1,2,4
	No stuffed animals allowed	4
	Always keeping your room clean	4
	Food allergy	3
	Can't go to the zoo	2
	Allergy in general	1,2,3,4,5
	Triggers in general	1,2,3,4,5
*Physical complaints including asthma symptoms*		

	**Cough**	1,2,3,4,5
	**Shortness of breath**	1,2,3,4,5
	**Wheezing**	1,2,3,4,5
	Other physical complaints:	
	*Being tired	4,5
	*Having a red head	1
	*Headache	2,3
	*Stomach-ache	2
	*Pain	3
	*Being sick	2,3
	*dizziness	2
	**Sore throat**	1,2,3,5
	Night time complaints	1,2,3,4
	**Asthma attack**	1,2,5
	How bad is asthma	3,4,5
	Sputum production	2,4
	Out of breath/deep breath	3
	Difficult to laugh	1
*Limitations in activities*		

	**Running**	1,2,3,4,5
	**Sport-activities, like cycling**	1,2,3,4,5
	Can not keep up with others/feeling less capable	1,2,3,5
	**Swimming**	1,2,3,4,5
	**Physical education**	1,2,3,5
	Playing outside	2,3,5
	Carrying heavy stuff	2
	Being busy	2,4
*Impact on social life*		

	**Being bullied, left out and not believed**	1,2,3,4,5
	**Visits to doctors, hospitals, tests**	1,3,5
	**Missing days at school**	1,3,5
	**Having to explain about asthma**	1,3,4,5
	Guilty feeling (asthma is annoying for others)	1,5
	Self management aspects	1,2,3,4
	Going to parties	2,4
	Playing	1,2,4,5
	Lack of consideration by others	1,2
	Limitations in family activities	1,3
	Having a friend with asthma (positive)	3,5
	Asthma camp (positive)	3
	Fail to meet the expectations of a parent	1
*Emotional and mental impact (including self management components)*		

	**Daily medication use**	1,2,3,4,5
	**Dependency on medication**	1,2,3,4,5
	**Difficult to concentrate/paying attention**	1,2,3,4,5
	**Feeling different (less popular) and lonely**	1,4,5
	Take medication with you	2,4,5
	Always have to take asthma into account	1,2,4
	Angry, hate, to be fed up	1,4,5
	Feeling sad	1,5
	Fear for asthma attacks and dyspnoea	1,5
	Take medication in front of others	2,4,5
	Frustrated	4
	Worried/concerned or troubled	4
	Peak flow measures	1,4
	Fear about the future	4

^1 ^Components in the table that were selected as essential components of asthma-specific HRQL are displayed in bold.

**Table 3:** Overview of number of statements expressed by paediatric asthma patients per component of HRQL.

Domain	Component	Number of statements by children of the focus groups
*Limitations due to environmental triggers*		

	No pets^1^	28
	Cigarette smoke	21
	Dust (house dust mite)	18
	Seasonal changes	13
*Physical complaints including asthma symptoms*		

	Cough	49
	Shortness of breath	47
	Wheezing	34
	Sore throat	10
	Asthma attack	8
*Limitations in activities*		

	Running	39
	Sport-activities, like cycling	29
	Swimming	16
	Physical education	11
*Impact on social life*		

	Being bullied, left out and not believed	60
	Visits to doctors, hospitals, tests	32
	Missing days at school	22
	Having to explain about asthma	11
*Emotional and mental impact (including self management components)*		

	Daily medication use	22
	Dependency on medication	22
	Difficult to concentrate/paying attention	17
	Feeling different (less popular) and lonely	11

^1 ^Components in the table are selected as essential components based on the outcome of the focus group meetings

After scoring the separate components, use of medication, exposure to cigarette smoke, being short of breath, and being bullied by peers ranked highest on the list of negative components of HRQL based on NGT. An overview of all components mentioned by the children is given in Table 4.

**Table 4:** Most important components of HRQL according to asthmatic children; results of the nominal group technique

Component of HRQL	Total score^1^
Need to use medication	18
Effect of cigarette smoke	12
Shortness of breath	9
Being bullied by peers	9
Cough	7
Limitations due to allergic triggers (in general)	7
Unable to have a pet	5
Lack of concentration	4
Limitations in running	3
Limitations school gymnastics	3
Asthma attacks	3
Limitations in swimming	2
Limitations in sport activities in general	2
Doctor visits	2
Missing days at school	2
Being angry	2
Being sick	2
Hospital visits	1
Weather influence	1

^1 ^Children were asked to select a maximum of three worst components of asthma-specific health-related quality of life. The most important component according to a child received 3 points, the second most important component received 2 points, and the third most important component received 1 point. A total score per component was calculated.

### Narrative description of the components and domains of HRQL

The data description was generally comparable between the two reviewers, and the two developmental psychologists agreed with the reviewers' conclusions. The third reviewer was consulted on one discussed component: medication use. In Figure 1, the domains on HRQL are presented, including the most important components according to the children. Although a wide range of components on HRQL were mentioned, it became clear that these components were considered to be vital for the children. As shown in Figure 1, domains and components of HRQL are related and interact with other components and domains.

### Limitations due to environmental triggers

The most frequently mentioned trigger that resulted in social limitations according to the children was exposure to environmental cigarette smoke. Moreover, children disliked the fact that they were not allowed to keep a furry pet or to caress such a pet. Due to seasonal changes, children experienced the difference between periods with relative mild symptoms compared to periods with more frequent or severe symptoms. Finally, the avoidance of house dust resulted in important limitations according to the children, like no permission to play at a dusty attic.

#### Illustrative quotes regarding limitations due to environmental triggers

"Because we have two attics and in one of them we might be allowed to build a hut and then I can hardly go in it."

"Yes, because mummy and daddy, they do that a lot (smoking) and then I have to go to the living room, because daddy and mummy are doing it in the kitchen."

"I can't play with furry animals, and we don't have a dog either but daddy does want one."

### Asthmatic symptoms

Wheezing, dyspnea, and coughing were frequently mentioned symptoms. Waking up at night was not an important issue according to the interviewed children. In contrast, the children considered having a sore throat as detrimental. When a child had experienced an acute exacerbation, this had a tremendous impact on his or her life. Symptoms were related to limitations in activities and have social implications (like not been able to run long enough to score a point in a soccer game or classmates that react annoyed on the child's wheezing).

#### Illustrative quotes of the children regarding asthmatic symptoms

(About hospital admission due to exacerbation). "Yes, not very nice. Because it was Sinterklaas, that was last year. And that was in the middle of the night and then I asked mummy if it was a dream, because I didn't know if it was a dream or not." Note: Sinterklaas is a Dutch children's festival with key figure Sinterklaas who brings presents

"Yeah, I have that sometimes, I cough all night long. But daddy and mummy are disturbed more than I am."

### Limitations in activities

Being limited in activities was an important component of HRQL according to the asthmatic children. The main physical activity that was limited due to asthmatic disease was running. Being able to run fast influences success in many games and activities. Moreover, the limitations in physical capacities which resulted in being less good in sports, like swimming and cycling were of concern to the asthmatic children.

#### Illustrative quotes of the children regarding activity limitations

"I have it with gymnastics and water gymnastics, you have to run in circles and halfway round I have to cough a lot so I usually have to sit aside because then, otherwise it's annoying for the others."

"I try to run as fast as possible at the start and then I try to keep up. But I fall further and further behind. And everyone says like: "come on, faster". And then, well, that's about it.. .. that I have to run faster."

### Impact on social life

The children frequently mentioned being bullied or ignored because of their limited physical capacities, especially during physical education at school. For instance, in the formation of teams, the asthmatic children felt they were less likely to be chosen. Moreover, a slower child is an easy target in some games (like tag or hit ball). The inevitable visits to health care professionals or a hospital and missing school activities due to these visits and illnesses were also considered to be negative consequences by some of the asthmatic children. Next to the unpleasant diagnostic procedures, like histamine provocation tests, and feeling ill, children felt they missed important schooling and found it hard to keep up with the class. Moreover, the children frequently had to face classmates' disbelief that they considered school absence as a negative and not a positive thing. Asthmatic symptoms like coughing cannot always be kept hidden for classmates and could result in bullying. Having to explain aspects of asthma over and over again was another aspect of the disease the children disliked. Most children did not experience problems in the relationship with their parents and felt that they were not treated differently than their siblings.

#### Illustrative quotes of the children regarding the impact on their social life

"And like once, I had to go to hospital and then I went to school and then they all said "liar, you didn't have to go to hospital and so", and it WAS true because I did have to go to hospital."

"Everyone thinks that I, then I have to go home during school hours because I'm ill and then everyone thinks yeah she doesn't have to do maths, then they think asthma is fun, but it isn't fun at all. I'd rather be at school than ill."

"I do have it sometimes, then I have to cough during class and then on the playground they, they bully me and such. That I did that."

### Emotional and mental impact

The children emphasized that they felt different from peers. Medication use was a negative aspect of asthmatic disease that was frequently mentioned. However, the interviewed children were thinking rather positively about the future. They expected that improved medication might become available in the future, that bullying would develop to be less frequent at high school, and hoped that they would outgrow the disease like they had heard from some people. The most frequently mentioned cognitive complaint was the lack of concentration in school. Coughing, in particular, was found to disturb their concentration.

#### Illustrative quotes regarding emotional and mental impact

"No, because when I get out of bed like that, and then I have to take medicine and then I'm like, yeah then I sometime, I really don't want to, but I do have to. And that takes a while. And when I have to, I say to myself (colloquial), only when I don't have to, then I'd be happy. Because sometimes in class, I have to cough, but not often, but it's not nice. Uh yeah."

"Well sometimes I feel a bit different when I suddenly have trouble with my lungs."

"(About future:) Yes and when they bully you now, WHEN, the pushing when you can't run so fast, when you're older it won't be like that."

## Discussion

In this qualitative study, components of HRQL as experienced by children with asthma themselves were explored. The most important components of HRQL were the consequences and negative effects of asthma on peer relationships (e.g., being bullied), the dependence on medication, shortness of breath, cough, limitations in activities, and the social limitations as a result of having to avoid environmental cigarette smoke.

### Comparison with existing asthma-specific HRQL questionnaires

Most components of HRQL according to the participants in our focus group sessions are also part of at least one of the four most prominent questionnaires (i.e., PAQLQ, HAY, PEDsQL, and CAQ-B). Components from our study that are not part of these questionnaires are: sore throat and triggers other than cigarette smoke. A sore throat is not a direct effect of asthmatic disease but could be a side effect of cough and/or the use of inhaled corticosteroids. In contrast, waking up at night is an item in all questionnaires but was not an issue according to the children in our focus group sessions. All items of the existing childhood asthma-specific HRQL questionnaires were specifically explored if the children did not spontaneously mention these components. Also, when the moderator probed waking up at night, the majority of children declared that this was not an important issue. Environmental triggers were not part of any of the four questionnaires, except for not being allowed to caress a pet. That environmental triggers can influence the health-related quality of life of children with asthma has been emphasized recently [21].

There is major disagreement between the four HRQL questionnaires on components of asthma-specific HRQL. One possible explanation for this result is that the various childhood asthma-specific HRQL questionnaires cover different aspects of asthma-specific HRQL. In some cases, the exclusion of specific components was intentional. For example, it was the decision of the developers of the PAQLQ to exclude medication use because of the major impact medication use can have on the overall HRQL score [22]. An additional explanation may be the different item-selection procedures that were used to develop the four questionnaires. For the development of the CAQ-B, focus group sessions with children (not all asthmatic) were held, next to workshop meetings with health care professionals [23]. For the development of the PAQLQ, children with asthma and their parents were asked to identify the important components on a list of possible items that had been pre-selected by experts, literature, and eight asthmatic children [24]. The HAY instrument was mainly developed based on expert opinion [25]. The item selection for the original PEDsQL™ was based on a literature search, interviews with cancer patients and their families, and discussions with pediatric health care professionals [26].

For scientific research, we would recommend to use the PAQLQ, though it does not surpass the other instruments in the agreement with our model. Still, the PAQLQ is the most frequently used instrument, and therefore, using this instrument has the benefit for researchers that results could more easily be compared with previous findings. Moreover, there is a version of the PAQLQ that enables children to select personal activities instead of standardised activities. Therefore, activities evaluated with this version of the PAQLQ are certainly relevant for the child that fills out the questionnaire.

### Limitations of the study

The primary reason to stratify children on age and disease severity was to gain maximum variation in the study population on these aspects. Moreover, children were stratified to enhance free-flowing conversations. Although maximum variation was achieved, it was not possible to stratify the children as planned in groups, since it was important that the focus group meetings took place in the neighborhood of the participants and the locations were rather widespread. In general, the moderators observed no negative influence of the composition of the groups on the discussion. Also, aspects like gender, social economic status, and family structure could have influence on the HRQL of children, but maximum variation on all these aspects was found in the focus group population (table 1). Moreover, in focus group research, only a small number of responders are participating, which may be a limitation for the generalization to a larger population [27]. Our study took place in the Netherlands, and though it can be that some findings are influenced by cultural habits or local laws (e.g., at the time of the study there was no smoking ban for bars and restaurants in the Netherlands), we believe that the components of HRQL mentioned by the children are relevant for children with asthma in general. Finally, many children invited by general practitioners did no longer suffer from asthmatic complaints, according to the parents. Most likely, children were classified with asthma earlier in life while their respiratory complaints were transient.

### Clinical implications

Some of the aspects of asthma-specific HRQL, according to the children, like being bullied and exposure to cigarette smoke, can be altered. For that reason, gathering information on HRQL in daily care with a valid HRQL instrument may not only contribute to better insight in the influence of asthma on the child's life, but also has the potential to improve the HRQL of the pediatric asthma patient, since the components of HRQL that are bothering the pediatric asthma patient can be integrated in medical care decisions for the individual asthmatic child. Using the existing HRQL instruments in daily care may have some important disadvantages. There is major disagreement between the instruments; therefore, the conclusion will depend on the chosen instrument. Moreover, some essential components are missing in all instruments (e.g., avoiding environmental cigarette smoke). More importantly, the standardization of all existing HRQL instruments for childhood asthma results in loss of valuable information on the HRQL of an individual child. With an individualized HRQL instrument for childhood asthma, these negative aspects could be avoided [28]. Based on the outcome of the focus group meetings, we are going to develop an individualized HRQL instrument for childhood asthma.

## Conclusion

The most important finding from this qualitative study was that asthma influences the life of children physically, emotionally, and socially. The most important components of HRQL were the consequences and negative effects of asthma on peer relationships (e.g., being bullied), the dependence on medication, shortness of breath, cough, limitations in activities, and the social limitations as a result of avoiding environmental cigarette smoke.

## Abbreviations

(CAQ-B): Childhood Asthma Questionnaire; (HAY): How Are You instrument; (HRQL): Health-related Quality of Life; (NGT): Nominal group technique; (PAQLQ): Pediatric Asthma Quality of Life Questionnaire; (PEDsQL™): Pediatric Quality of Life Inventory.

## Competing interests

Lisette van den Bemt, Sabine Kooijman, Vinca Linssen, Jean Muris, and Gordon Slabbers have no conflicts of interest to disclose; Tjard Schermer received grant money for research in the field of respiratory medicine from non-commercial organizations (Radboud University Nijmegen Medical Centre, the Netherlands Organization for Health Research and Development (ZonMw), and the Dutch Asthma Foundation), and from several pharmaceutical companies (Boehringer Ingelheim, AstraZeneca, and GlaxoSmithKline).

## Authors' contributions

LB and SK designed the study and analysed the data. VL, PL, JM and GS were participants of the scientific research committee that assisted in the development of the research protocol and focus group route. SK, PL and GS were involved in the selection of participants. LB and TS drafted the manuscript and all authors participated in the discussion and interpretation of the final results, contributed to the final paper, and approved the final version submitted for publication. The authors take responsibility for the data integrity. TS supervised the study and LB is guarantor.
